# Fatal Neutropenia Sepsis Following Acute Methotrexate Toxicity

**DOI:** 10.7759/cureus.77754

**Published:** 2025-01-21

**Authors:** Nicholsan Jesiah, Yathukulan Siva, Sundaresan KT

**Affiliations:** 1 University Medical Unit, Teaching Hospital - Batticaloa, Batticaloa, LKA; 2 Clinical Medicine, Faculty of Health Care Sciences, Eastern University, Teaching Hospital - Batticaloa, Batticaloa, LKA

**Keywords:** granulocyte-macrophage-colony-stimulating factor (gm-csf), methotrexate, psoriasis, rheumatoid arthritis, toxicity

## Abstract

Methotrexate (MTX) is widely used to manage autoimmune diseases such as rheumatoid arthritis, psoriasis, and eczema due to its anti-inflammatory and immunosuppressive properties. We present the case of a 69-year-old male individual with stage 3b chronic kidney disease (CKD) and diabetes mellitus, who developed acute MTX toxicity following a dosing error. The patient, prescribed MTX 7.5 mg weekly for chronic lower leg eczema, mistakenly took 5 mg every eight hours for four days. He presented with erythematous rashes, oral mucosal ulcerations, and pancytopenia, later diagnosed as neutropenic sepsis. Management included discontinuation of MTX, intravenous folinic acid, granulocyte-macrophage colony-stimulating factor (GM-CSF), and broad-spectrum antibiotics. By day 7, his blood counts and symptoms improved, and he was discharged on day 12. This case underscores the importance of patient education on proper MTX dosing to prevent life-threatening complications, particularly in high-risk populations such as the elderly and those with CKD.

## Introduction

Methotrexate (MTX) is a folic acid antagonist widely used in oncology and for managing chronic inflammatory and autoimmune diseases such as rheumatoid arthritis, psoriasis, and eczema [1]. It inhibits cellular proliferation, particularly affecting rapidly dividing cells, which accounts for its therapeutic benefits and potential toxicities. Common adverse effects include mucositis, cytopenia, and increased risk of infections, with toxicity risk heightened in specific populations [2].

MTX is primarily eliminated by the kidneys, with 80%-90% excreted unchanged in the urine. Patients with chronic kidney disease (CKD) are at greater risk of toxicity, even at low doses, due to impaired drug clearance. Clinical guidelines emphasize starting MTX at lower doses in such patients [3]. However, incorrect dosing or mismanagement, especially in elderly patients with multiple comorbidities, can lead to serious complications like pancytopenia and neutropenic sepsis.

Approximately 1.4% of patients on low-dose MTX develop pancytopenia [4], often triggered by errors in administration or inadequate monitoring [5]. Neutropenic sepsis, a life-threatening complication of pancytopenia, carries a high mortality rate if not promptly treated. Here, we present the case of a 69-year-old male individual with CKD and chronic eczema, who developed pancytopenia and neutropenic sepsis following an accidental MTX overdose, highlighting the critical importance of patient education and monitoring in MTX therapy.

## Case presentation

A 69-year-old male individual with a history of non-insulin-dependent diabetes mellitus, stage 3b chronic kidney disease (CKD), and chronic eczema on both feet presented with generalized skin rashes and painful oral mucosal sores for one week. He had mistakenly taken methotrexate (MTX) 5 mg every eight hours for four days instead of the prescribed weekly dose of 7.5 mg, which was initiated for eczema management. The patient had been on oral hypoglycemic medications and regularly attended medical clinic follow-ups.

**Figure 1:**
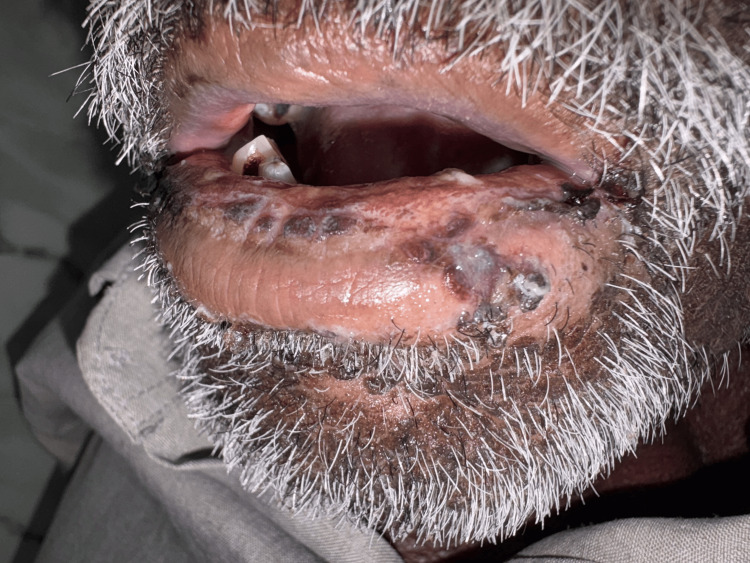
Oral mucosal inflammation with bleeding and ulceration.

**Figure 2:**
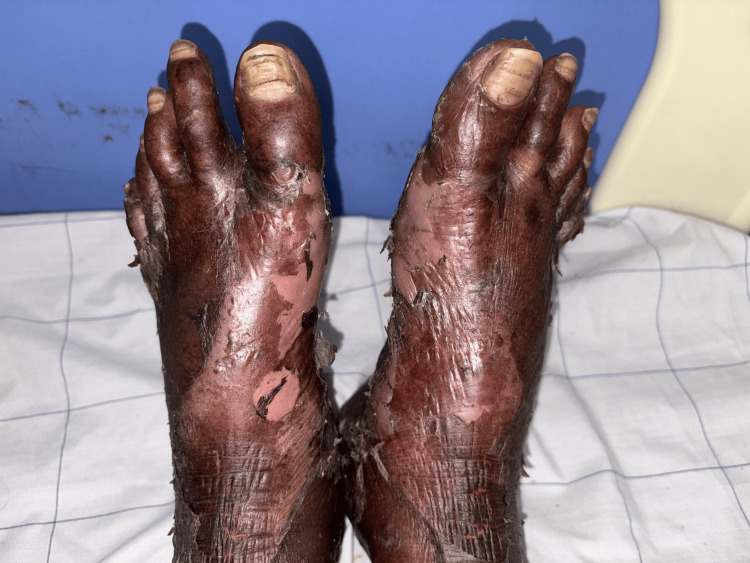
Erythematous lesions over the lower limb with scaling and ulceration.

On admission, the patient was conscious and oriented, with stable vital signs (blood pressure 115/70 mmHg, pulse rate 92 beats per minute, respiratory rate 16 breaths per minute, temperature 99.5°F, and oxygen saturation of 99% on room air). Examination revealed pallor, large painful ulcerative lesions with bleeding in the oral cavity (Figure 1), and generalized erythematous scaly rashes with ulcerative lesions, most prominent on the lower limbs (Figure 2). Systemic examination findings were unremarkable.

Laboratory investigations showed Impaired renal function (eGFR {estimated glomerular filtration rate} 31 mL/min/1.73m²), pancytopenia (neutrophils 0.06×10³/µL, hemoglobin 8.2 g/dL, and platelets 12×10³/µL), elevated inflammatory markers (CRP {C-reactive protein} 424 mg/L, procalcitonin 56 ng/mL, and ESR {erythrocyte sedimentation rate} 125 mm/hr) but liver function tests were normal. Cultures from blood, urine, and wound swabs were sterile, and screening for HIV, hepatitis B, and hepatitis C was negative. Table 1 presents a summary of the relevant investigations.

**Table 1:** Summary of investigations. AST: aspartate aminotransferase, ALT: alanine aminotransferase, CRP: C-reactive protein, ESR: erythrocyte sedimentation rate, INR: international normalized ratio, KUB: kidneys, ureters, and bladder, MTX: methotrexate.

Test (unit)	Value	On discharge	Normal value
WBC (*10^3^/uL)	0.47	11.1	4-11
Neutrophil (*10^3^/uL)	0.06	75.3	2-7
Hemoglobin (g/dL)	8.2	9.5	12-16
Platelet (*10^3^/uL)	12	100	150-450
AST (U/L)	31		15-37
ALT (U/L)	41		12-78
Serum Creatinine (micmol/L)	200		62-115
CRP (mg/L)	424	48	0-5
ESR (mm/1^st ^hour)	125	55	<20
Serum sodium (mmol/L)	142		136-145
Serum potassium (mmol/L)	3.5		3.5-5.1
INR	1.2		<1.2
Blood culture & Urine culture	Negative		
Ultrasound scan of abdomen & KUB	Features of chronic kidney disease only (poor corticomedullary demarcation)		
Blood picture	Pancytopenia due to possible MTX toxicity		

**Figure 3:**
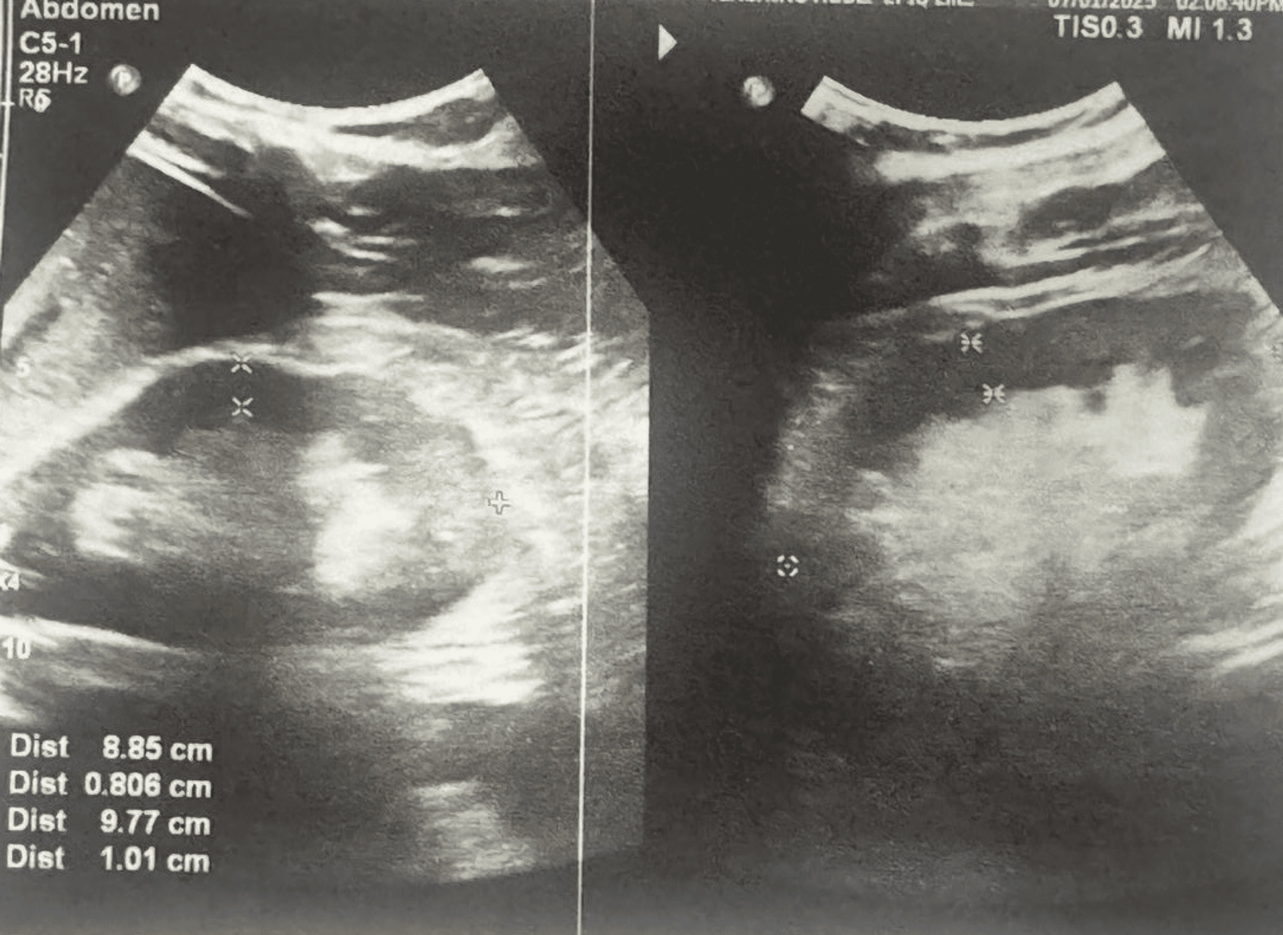
Bilateral kidneys show hyperechoic renal cortex with altered corticomedullary demarcation favors chronic kidney disease.

Abdominal ultrasound findings were consistent with chronic kidney disease (Figure 3).

A diagnosis of severe neutropenia sepsis secondary to MTX-induced bone marrow suppression was made. On day 2, the patient was transferred to the high-dependency unit (HDU) and managed with intravenous piperacillin-tazobactam (4.5 g every eight hours), oral fluconazole (100 mg weekly), intravenous folinic acid (15 mg every six hours) and subcutaneous granulocyte-macrophage colony-stimulating factor (G-CSF) (300 MU/day). Supportive care was given including analgesics, antihistamines, intravenous fluids, multivitamins, oral antivirals, and topical applications (liquid paraffin, fusidic ointment, chlorhexidine mouthwash, and local lignocaine) while maintaining the reverse barrier method.

By day 7, the patient’s blood counts and symptoms improved significantly, with mucosal bleeding and skin lesions resolution. He was discharged on day 12 with a follow-up arranged with the dermatologist for ongoing eczema management.

## Discussion

MTX, in low weekly doses, is a first-line treatment for chronic inflammatory diseases and certain cancers due to its anti-inflammatory and immunosuppressive properties [6]. Despite its benefits, MTX can cause severe adverse effects, including pancytopenia, mucositis, and cutaneous ulcerations, especially in high-risk populations. Risk factors for MTX toxicity include advanced age, CKD, hypoalbuminemia, infections, and polypharmacy [7,8]. Among these, CKD is a critical factor due to impaired drug clearance, which prolongs exposure and exacerbates toxicity.

In this case, the patient’s inadvertent daily dosing of MTX, combined with the absence of folic acid supplementation and underlying CKD, led to acute toxicity. MTX-induced pancytopenia and mucositis were prominent, with the latter contributing to decreased oral intake and prerenal acute kidney injury, further worsening renal function. These findings underscore the importance of identifying predisposing factors when prescribing MTX.

Management of MTX toxicity involves immediate drug discontinuation and initiation of supportive care [9,10]. In this patient, intravenous folinic acid and granulocyte-colony stimulating factor (G-CSF) effectively managed hematological toxicity. Broad-spectrum antibiotics addressed the risk of neutropenic sepsis, a life-threatening complication [10]. Although serum MTX levels were not measured, clinical signs strongly suggested toxicity, reinforcing the need for early intervention based on presentation rather than laboratory markers.

This case highlights several critical learning points, which are CKD and hypoalbuminemia increase the risk of MTX-induced bone marrow suppression by enhancing drug exposure [3], early signs of MTX toxicity such as mucositis, which has led to decreased oral intake causing prerenal acute kidney injury which worsened his renal function and proper patient counseling and regular follow-up are essential to prevent dosing errors, especially in elderly patients with multiple comorbidities.

Preventing MTX toxicity requires a multidisciplinary approach, including tailored dosing, education on medication use, and vigilant monitoring for early signs of complications. Comparisons with similar cases emphasize that adherence to clinical guidelines and proactive measures can minimize morbidity and mortality associated with MTX therapy.

## Conclusions

Methotrexate (MTX) is a valuable medication for managing autoimmune and inflammatory conditions when used appropriately. However, this case highlights the potential for severe toxicity, even at low doses, when dosing errors or predisposing factors such as chronic kidney disease and advanced age are present. Acute MTX toxicity can lead to life-threatening complications like pancytopenia and neutropenic sepsis, underscoring the need for careful monitoring and individualized dosing.

This case emphasizes the critical importance of thorough patient counseling to ensure a proper understanding of the dosing schedule and potential risks. Regular follow-ups and adherence to clinical guidelines are essential to prevent avoidable errors and reduce morbidity and mortality associated with MTX therapy. By prioritizing patient education and monitoring, clinicians can minimize adverse outcomes and enhance the safe use of this effective but potentially hazardous drug.
